# Long-term results after non-operative and operative treatment of radial neck fractures in adults

**DOI:** 10.1186/s13018-018-0731-3

**Published:** 2018-02-02

**Authors:** Holger Keil, Marc Schnetzke, Arpine Kocharyan, Sven Yves Vetter, Nils Beisemann, Benedict Swartman, Paul-Alfred Grützner, Jochen Franke

**Affiliations:** Clinic for Trauma and Orthopaedic Surgery, BG Trauma Center Ludwigshafen at Heidelberg University Hospital, Ludwig-Guttmann-Strasse 13, 67071 Ludwigshafen, Germany

**Keywords:** Radial neck fracture, Elbow, Mayo Elbow Performance Score, DASH score

## Abstract

**Background:**

The aim of this study is to determine the functional long-term outcome after non-operative and operative treatment of radial neck fractures in adults.

**Methods:**

Thirty-four consecutive patients with a mean age of 46.4 (18.0 to 63.0) years with a fracture of the radial neck who were treated between 2000 and 2014 were examined regarding the clinical and radiological outcome. Twenty patients were treated non-operatively, and 14 patients underwent surgery.

**Results:**

After a mean follow-up of 5.7 (2.0 to 15.7) years, the clinical scores showed good results in both groups. The Disabilities of Arm, Shoulder and Hand score was 16.1 (0 to 71.6) in the non-operative group and 8.8 (0 to 50.8) in the operative group, respectively. The Mayo Elbow Performance Score was 80.0 (30 to 95) in the non-operative group and 82.5 (35 to 95) in the non-operative group, respectively. The initial angle of the radial head towards the shaft (RHSA) was significantly higher in the operative group in the anterior-posterior plane (12.8° [2 to 23] vs. 26.3° [1 to 90], *p* = 0.015). In the follow-up radiographs, the RHSA was significantly lower in the operative group (15.1° [3 to 30] vs. 10.9° [3 to 18], *p* = 0.043). Five patients developed 7 complications in the non-operative group, and 7 patients developed 12 complications in the operative group. Revision rates were higher in the operative groups as 1 patient received radial head resection in the non-operative (5%) group while 7 patients in the operative group (50%) needed revision surgery.

**Conclusion:**

A good functional long-term outcome can be expected after operative and non-operative treatment of radial neck fractures in adults. If needed due to major displacement, open reduction is associated with a higher risk of complications and the need for revision surgery but can achieve similar clinical results.

**Trial registration:**

DRKS DRKS00012836 (retrospectively registered)

## Background

Isolated fractures of the radial neck are a rare injury of the elbow in both adults and children. In children, they account for about 5–10% of all elbow injuries [1, 2]. The radial neck fractures in children are classified according to Judet as modified by Maitezeau in types 1–4, according to the initial grade of displacement [3]. In dislocated fractures (initial angulation > 30°, according to type 3 and above), operative treatment is recommended due to the increasing risk of avascular necrosis [1, 2, 4–6].

In adults, fractures of the radial neck are even rarer still [7]. At present, very little data is available regarding the clinical and radiological outcome of these fractures in adults, especially when treated surgically [8, 9]. Radial neck fractures in adults can be classified according to Broberg-Morrey [10], which is a modification of the Mason classification [11]. A majority of isolated radial neck fractures, especially types 1 and 2 according to the Broberg-Morrey classification, can be treated by functional exercises alone, achieving excellent or good results in most patients [8, 12–14]. In comminuted and dislocated fractures of the radial neck, operative treatment with open reduction and internal fixation with screws or plate osteosynthesis is common [15–17]. In the available literature, results of radial neck fractures in adults are often evaluated and discussed together with radial head fractures [8, 9, 15–18]. Therefore, specific recommendations for treatment decision, especially in isolated radial neck fractures, are currently not available.

In the present study, the functional and radiological outcome of non-operatively and operatively treated fractures of the radial neck in adults has been investigated in detail. The primary aim was to evaluate the clinical and radiological long-term outcome of these fractures. The secondary aim was to evaluate the risk of complications and revision surgery following the initial treatment decision.

## Methods

### Study population

This retrospective cohort study was conducted after approval by the local ethics committee (Committee of the Medical Association of Rhineland-Palatinate, Mainz, Germany, reference no. 837.261.15). Patients that were treated with a radial neck fracture between 2000 and 2014 were recruited from the institutional register. Inclusion criteria were (1) age of 18 years or older at the time of the accident, (2) isolated fracture of the radial neck type 1 to 3 according to Broberg-Morrey [10] and (3) radiological and clinical minimum follow-up of 2 years.

Patients with a dislocation of the elbow or injuries of the ligamentary complex of the elbow were excluded. Finally, 34 consecutive patients with a mean age of 46.5 ± 13.6 years could be included. The patients were separated into two groups (non-operative and operative) according to the initial treatment decision.

### Treatment and rehabilitation

**Fig. 1:**
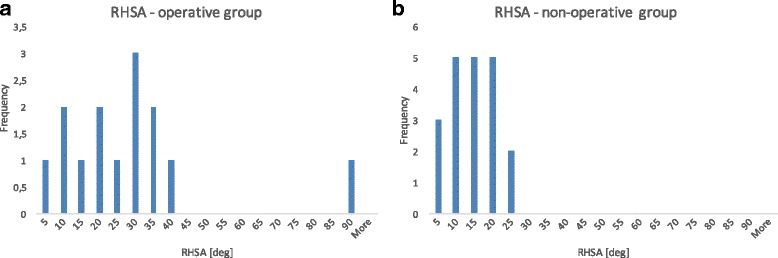
**a** Preoperative CT scan and intra-operative fluoroscopy of crossed screw osteosynthesis. **b** Preoperative X-ray and intra-operative fluoroscopy of plate osteosynthesis

Treatment decision for operative or non-operative treatment was taken on an individual basis depending on degree of fracture displacement, age, involved side, state of activity, profession and patient’s individual preference. In the case of operative treatment, a radial incision was performed and care was taken not to release the lateral ligament complex. The annular ligament was intersected to expose the radial neck fracture. Screw and/or plate osteosynthesis (Depuy-Synthes, West Chester, USA, Fig. 1) was performed to stabilise the fracture with special care taken to positioning the implant in the safe zone of the proximal radius [19–22]. Finally, the correct implant placement and fracture reduction were checked under dynamic fluoroscopy. Full supination and pronation without impingement of the implant was ensured by intraoperative visualisation and by fluoroscopy. Afterwards, the annular ligament and the capsule were sutured.

Irrespective of the treatment group, all patients underwent temporary plaster immobilisation in a posterior splint at 90° of elbow flexion for 1 week. According to the rehabilitation protocol, functional treatment began within 7 days after trauma at the latest with exercises in a pain-free range of motion under avoidance of forearm rotation and axial stress during the first 6 weeks.

### Clinical assessment

Clinical outcome was assessed by the Disability of the Arm, Shoulder and Hand (DASH) score, Mayo Elbow Performance Score (MEPS), range of motion (ROM) in extension/flexion of the elbow as well as in pronation and supination, pain in rest and motion according to the visual analogue scale (VAS), duration of inability to work and pre- and posttraumatic activity in sports. Quality of life was assessed using the Short Form Health Survey (SF-36) questionnaire. Extension deficit was defined as the lack of extension towards the non-injured side.

### Radiographic evaluation

**Fig. 2:**
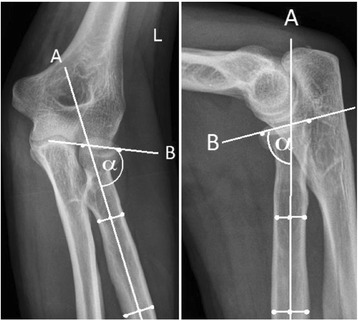
Method to determine the dislocation of the radial head towards the radial shaft. First, a line following the axis of the radial shaft is constructed by determining the centre of the radial shaft in two locations (line A). Second, a line is created by connecting the edges of the radial head (line B). The angle alpha between the two lines is the angle of the radial head towards the radial shaft. The radial head-shaft angle is defined as the opposite angle to alpha

The fractures were classified according to the Broberg-Morrey modification of the Mason classification [10]. To determine the influence of initial fracture displacement on clinical outcome, the angulation of the radial head towards the radial shaft was measured in anterior-posterior and lateral views in the initial posttraumatic radiographs as well as in the postoperative follow-up radiographs (radial head-shaft angle; RHSA). The measurement procedure for the determination of the RHSA is illustrated in Fig. 2. The measurement procedure was performed by blinded examiners [SV and BS]. The follow-up radiographs were used to assess complications such as pseudoarthrosis and failure of fracture fixation.

In addition, complications and the need for revision surgeries were assessed. Pain with VAS in motion > 5 and limitation of extension/flexion of > 35° compared to the healthy side were defined as complications.

Descriptive statistics (mean, standard deviation, absolute and relative frequencies) are reported for the characterisation of the study population. The Mann-Whitney *U* test was used for statistical analysis. A *p* value of < 0.05 was considered to show a significant difference.

## Results

**Fig. 3:**
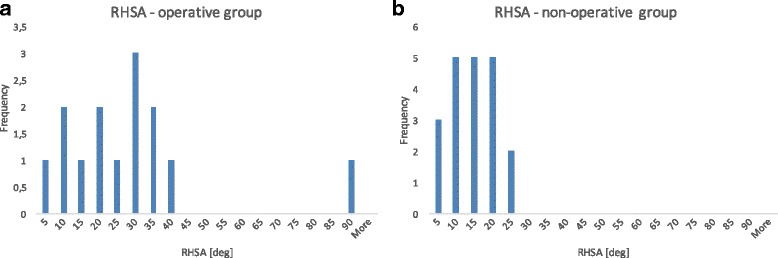
**a** Histograms of the distribution of the initial radial head-shaft angle (RHSA) in the anterior-posterior plane in the non-operative group. **b** Histograms of the distribution of the initial radial head-shaft angle (RHSA) in the anterior-posterior plane in the operative group

Demographic data of the study population with variation of the treatment groups are shown in Table 1. Twenty patients (58.8%) were treated non-operatively; 14 patients (41.2%) received primary operative treatment. Four patients of the operative group (28.6%) were treated by crossed screw osteosynthesis, 10 patients (71.4%) by plate osteosynthesis. The mean follow-up was 5.7 (2.0 to 15.7) years (non-operative group 5.6 [2.0 to 15.7], operative group 5.7 [2.1 to 12.7] years). The distribution of the fractures according to the Broberg-Morrey classification showed that patients in the non-operative group had less severe injury types (Table 2). The initial RHSA was larger in the operative group with a significant difference in the anterior-posterior plane (*p* = 0.015 (Fig. 3, Table 3). At postoperative radiographs, the RHSA was significantly lower in the operative group (*p* = 0.043). Due to the significant difference of the RHSA in the initial radiographs, statistical tests for the comparison of the clinical results of the two groups were not performed. Regression analysis of the initial RHSA regarding the DASH score and MEPS outcome was performed, regarding a correlation between angulation and functional results. There was no strong correlation between RHSA and DASH (*R* = 0.253) or MEPS (*R* = 0.205).

**Table 1:** Demographic data with variation of the study groups

	Non-operative (*n* = 20)	Operative (*n* = 14)
Age [years]	47.6 (22 to 63)	44.9 (18 to 63)
Gender [%]		
Male	11 (55)	5 (35.7)
Female	9 (45)	9 (64.3)
Affected side [%]		
Right	11 (55)	8 (57)
Left	9 (45)	6 (43)

**Table 2:** Distribution of fracture types according to Broberg-Morrey modification of the Mason classification

Broberg-Morrey type	Non-operative (*n* = 20)	Operative (*n* = 14)
1	10 (50%)	0
2	9 (45%)	9 (64%)
3	1 (5%)	5 (36%)

**Table 3:** Radial head-shaft angle (RHSA) of the initial and follow-up radiographs

	Non-operative (*n* = 20)	Operative (*n* = 14)	*p* value
Initial [°]			
AP	12.8 (2 to 23)	26.3 (1 to 90)	0.012
Lateral	13.2 (2 to 33)	21.5 (2 to 90)	0.121
Follow-up [°]			
AP	15.1 (3 to 30)	10.9 (3 to 18)	0.043
Lateral	14.6 (2 to 33)	13.1 (3 to 24)	0.552

At follow-up, the overall clinical outcome was good with a mean DASH score of 13.1 (0 to 71.6) points and a mean MEPS of 81.0 (30 to 95) points. The detailed analysis of the clinical scores showed good results in both groups (Table 4). The quality of life assessed by the SF-36 was comparable to the average values of the normative groups.

**Table 4:** Summary of the clinical scores after non-operative and operative treatment

	Non-operative (*n* = 20)	Operative (*n* = 14)
MEPS [pts]	80.0 (30 to 95)	82.5 (35 to 95)
VAS		
Rest	1.0 (0 to 5)	1.1 (0 to 5)
Motion	3.0 (0 to 10)	2.2 (0 to 7)
DASH [pts]	16.1 (0 to 71.6)	8.8 (0 to 50.8)
DASH (sports)	4.7 (0 to 25.0)	12.5 (0 to 50.0)
DASH (work)	13.0 (0 to 87.5)	19.4 (0 to 100)
SF-36 [pts]		
Physical health	49.5 (31 to 64)	50.8 (39 to 60)
Mental health	47.7 (16 to 61)	52.1 (27 to 64)
ROM [°]		
Extension deficit	6.2 (− 5 to 60)	3.2 (0 to 10)
Flexion	134.1 (115 to 145)	120.9 (90 to 140)
Pronation	84.1 (60 to 90)	74.5 (10 to 90)
Supination	81.8 (0 to 90)	80.0 (60 to 90)

Five patients (25%) developed a total of 7 complications in the non-operative group, and 7 patients (50%) developed a total of 11 complications in the operative group (Table 5).

**Table 5:** Summary of the complications with variation of treatment groups

No. of complications	Non-operative (*n* = 20, *Σ* = 5)	Operative (*n* = 14, *Σ* = 7)
VAS motion > 5	3	2
Restriction of ROM > 35°	1	2
Secondary displacement	1	5
Pseudoarthrosis	1	2
CRPS	1	0
Haematoma	0	1
Total	7	12

One patient (5%) received radial head resection in the non-operative group while 7 patients (50%) in the operative group underwent revision surgery (Table 6).

**Table 6:** Summary of revision surgeries with variation of treatment groups

No. of revision operations	Conservative (*n* = 20, *Σ* = 1)	Operative (*n* = 14, *Σ* = 7)
Implant removal	0	1
Radial head resection	1	2
Open arthrolysis	0	2
Re-osteosynthesis	0	1
Haematoma removal	0	1
Total	1	7

Two patients (10%) in the non-operative group did not reach ability to work, while all patients in the operative group were able to return to work. The mean time between the accident and return to work was 102.19 (31 to 319) days in the non-operative and 146.33 (28 to 459) days in the operative group.

Seventeen out of 20 patients (85%) in the non-operative group were doing sporting activities before the accident with a mean duration of 4.3 (1 to 13) hours per week. Seven out of 14 patients (50%) in the operative group were doing sporting activities with a mean duration of 8.4 (2 to 24) hours per week. At follow-up, 16 patients in the non-operative group (80%) were doing sporting activities with a mean duration of 4.1 (1 to 13) hours per week, while 6 patients in the operative group (40%) were doing sporting activities with a mean duration of 3.0 (0 to 8) hours per week.

## Discussion

Regardless of the treatment modality, it was possible to a show an overall good to very good functional long-term outcome of radial neck fractures regarding the clinical parameters DASH and MEPS. Range of motion and pain level also showed good results in both the operative and non-operative group at final follow-up.

A relevant number of the patients in the operative group suffered from complications and needed revision surgery. This can be assumed to be a consequence of common risks of surgery but also of this specific operative technique. As known from displaced radial head fractures that are operatively treated by plate osteosynthesis, it is crucial to position the plate in the safe zone (meaning the non-articulating portion of the radial head) to avoid plate impingement [20, 23, 24].

None of the 4 patients that were treated by crossed screw osteosynthesis needed revision surgery, while 6 of the 10 patients who received plate osteosynthesis needed revision surgery. This might be due to a higher complexity of these fractures with a multi-fragmentary situation. These results are in agreement of Li et al. who compared crossed screw osteosynthesis with plate osteosynthesis in radial neck fractures and found better results for the screw osteosynthesis [25].

The available literature often analyses the outcome of radial neck fractures together with fractures of the radial head. Duckworth et al. published a larger series of 237 patients who were suffering from a radial head or radial neck fracture and analysed the clinical outcome according to the MEPS after a mean follow-up of 6 months [8]. They recorded an excellent mean MEPS of 92 points, which is better than the results of the current study. They also reported of only two complications, which might be due to the rather short follow-up time. The revision surgery in our patients was performed after an average of 9.6 months. Most patients in this study were treated non-operatively.

Kang et al. published a follow-up of six patients that suffered from non-union after surgically treated displaced radial head and neck fractures [18]. All of them went well under non-operative treatment with a mean MEPS of 96.7 points at follow-up of 7.6 years. This is in contrast with the results of the present study, as two out of three patients with pseudoarthrosis needed revision surgery due to painful limitation of motion. Both patients were treated by excision of the radial head. The mean MEPS of these 3 patients was 78.3 points.

Herbertsson et al. published a long-term follow-up study (mean follow-up 19 years) including radial head and neck fractures Mason types 2 and 3 [12]. A minority of 2 of 100 patients received open reduction and osteosynthesis, while 19 patients were treated with radial head excision alone. The functional results were good with a tendency of impaired flexion of the injured side. In 2004, Herbertsson et al. published the results following radial head excision after radial head and neck fractures [26]. The functional outcome was overall fair to good with worse results of the initial Mason type 4 fractures. It should be noted, however, that a separate analysis of the radial neck fractures was not performed in these studies.

In summary, overall good results can be expected after non-operative and operative treatment of radial neck fractures with higher rates of revision surgery in the operative group. Nonetheless, the operatively treated patients with more severe injury types according to the Broberg-Morrey classification reached the same good clinical outcome parameters as the non-operatively treated patients at final follow-up. We therefore conclude that open reduction and osteosynthesis seem favourable for patients with comminuted and dislocated fractures.

There are several limitations of this study. First of all, the study is limited by its retrospective nature and its small sample size. Due to the rare incidence of this specific fracture, the observational groups are rather small. Secondly, the degree of angulation of the radial neck that led to the initial decision of operative or conservative treatment was significantly different in the two groups. This made a statistical testing between both groups impossible. The decision whether to treat non-operatively or operatively was made on an individual basis rather than by randomisation. Third, the radiological assessment of the initial angulation depends on the exact radiological technique. Due to the acute painful situation, some of the initial radiographs are not exactly anterior-posterior or lateral views. Furthermore, an interobserver and intraobserver analysis of the measurement method of the determination of the RHSA was not performed.

## Conclusions

Radial neck fractures are a rare entity in adults. Depending on the initial degree of angulation, a non-operative treatment can achieve good results in clinical scores. If open reduction and osteosynthesis is needed, crossed screw osteosynthesis seems to have a lower risk of surgery-associated complications. However, from the data of this study, it is not possible to determine a RHSA that necessitates open reduction. Further prospective studies might show a certain degree of RSHA that necessitates open reduction and osteosynthesis.

## Abbreviations

DASH: Disabilities of Arm Shoulder and Hand, a subjective score to evaluate the disabilities of the upper limb. Values range from 0 (no disability) to 100 (complete disability)
MEPS: Mayo Elbow Performance Score, a combined score with subjective and objective items (pain, motion, stability, function). Values range from 6 (poor) to 100 (excellent)
RHSA: Radial head-shaft angle, the angle between the articular surfaces of the radial head towards the radial shaft. This is used in this study to describe the amount of dislocation of the fractures
ROM: Range of motion, the zone in which the related joint is movable
SF-36: Short Form Health Survey, a score to scale the individual quality of life. The items are divided into eight sections that address physical and mental health. The result is compared to a normalised population that has a result of 50 points
VAS: Visual Analogue Scale, used to assess the individual pain level (0 = no pain, 10 = worst pain imaginable)
